# Discrepancies in the register of primary health care visits: a 6-year time series study from Finland

**DOI:** 10.1017/S1463423625100625

**Published:** 2025-11-18

**Authors:** Tuomas Majuri, Mikko Pussinen

**Affiliations:** 1 Research Unit of Population Health, University of Oulu, Oulu, Finland; 2 Medical Research Center Oulu, Oulu University Hospital and University of Oulu, Oulu, Finland; 3 Terveystalo Occupational Healthcare, Oulu, Finland; 4 Department of Surgery, Kanta-Häme Central Hospital, Hämeenlinna, Finland

**Keywords:** Diagnosis rates, equality, healthcare funding, healthcare registers, primary health care

## Abstract

Information on registered primary healthcare diagnoses from the Register of Primary Health Care Visits (RPV) is used to allocate healthcare funding in Finland. Our aim was to analyse the diagnosis rate trajectories in the RPV and, through that, assess the equitable development of funding. We extracted national- and regional-level diagnosis numbers from the RPV. Joinpoint regression analysis with Model 1 (overall trend) and Model 2 (potential changes in trend) was used to assess diagnosis rate trajectories from 2018 to 2024. Model 1 demonstrated that the number of registered primary healthcare diagnoses has increased between 2018 and 2024, but the growth has not been uniform across all trajectories. Model 2 showed significant differences in the diagnosis rate trajectories between regions and diagnostic groups. There were significant discrepancies in the registration of primary care diagnoses. Reducing these discrepancies by standardizing diagnosis registration practices is necessary to ensure equitable healthcare funding.

## Introduction

Healthcare registers provide valuable information for service planning and decision-making (Cheng, 2015; Laugesen *et al*., 2021, García‑Velázquez *et al*., 2022). Nordic healthcare registers are well known for their high-quality data (Mellander, 2017; Laugesen *et al*., 2021; Suokas *et al*., 2024). However, despite registers often undergoing studies on the validity and quality of their data (Sund, 2012), register data can have inaccuracies and gaps in coverage (Byford *et al*., 2007).

In Finland, several healthcare registers are used for various purposes (García‑Velázquez *et al*., 2022, Suokas *et al*., 2024). The specialized healthcare register, maintained by the Finnish Institute for Health and Welfare (FIHW), has been particularly utilized for research purposes due to its extensive coverage and accuracy (FIHW, 2025). The Register of Primary Health Care Visits (RPV) has been in use since 2011 to document all patient encounters in public primary healthcare (FIHW, 2025). In recent years, the use of the RPV has also increased, both in research and as a tool for societal decision-making (García‑Velázquez *et al*., 2022, Suokas *et al*., 2024). The coverage of RPV has increased but it is not yet as comprehensive as specialized healthcare register (FIHW, 2025). Despite poor coverage, primary healthcare register data is used in the planning of healthcare service funding at the national level between well-being regions (Holster *et al*., 2022) and in regional level between different sectors of healthcare (The Wellbeing Services County of North Ostrobothnia, 2025) in Finland.

The Finnish healthcare system is mainly based on public healthcare services (Ministry of Social Affairs and Health, 2025). The responsibility for providing healthcare services transferred to well-being regions in 2023. Care is provided in either primary healthcare or specialized healthcare, depending on individual service needs. Well-being regions can decide how to provide services within their region, within the limits of legislation. As a result, there may be regional differences in healthcare practices. The funding of well-being regions is based retrospectively on the data of the previous years of the respective region (Ministry of Finance, 2025). The funding for well-being regions is based on social and healthcare service need coefficients, which indicate the need for social and healthcare services in each area (Häkkinen *et al*., 2020; Holster *et al*., 2022). The calculation of need coefficients is based on diagnostic data, which also includes registered primary care diagnoses from the RPV (Häkkinen *et al*., 2020; Holster *et al*., 2022). A more detailed description of the formation of need coefficients and the funding model based on has been described elsewhere (Holster *et al*., 2022). The service provider’s funding increases when the population’s need for services, measured by the number of diagnoses, in the region rises (Häkkinen *et al*., 2020; Holster *et al*., 2022).

The quality of care should not vary based on geographic location or medical condition (WHO, 2025). For funding to be distributed equitably across different regions and healthcare sectors, registers used to assess the need for services must be accurate (Smith, 2008; Holster *et al*., 2022). The need for services should evolve consistently across regions and diagnostic groups, with changes driven by morbidity rather than differing practices (Smith, 2008). However, no previous studies have analysed this progression in relation to the RPV in Finland. The RPV has been criticized for inaccuracies in the recording of diagnostic data (Häkkinen *et al*., 2020; García‑Velázquez *et al*., 2022; Holster *et al*., 2022), raising concerns – also reflected in media discourse – regarding its reliability for use in healthcare funding planning. When planning funding, the use of variables that are strongly related to the activities of the service provider, such as service usage, may contribute to competition and result in an unequal allocation of funding (Smith, 2008; Holster *et al*., 2022).

Our aim was to analyse the diagnosis rate trajectories in the RPV and, through that, assess the equitable development of funding across well-being regions and diagnostic groups resulting from potential discrepancies in the registered need for services. The data included national- and regional-level register data on primary care diagnoses from 2018 to 2024.

## Methods

### Diagnosis rate trajectories

The purpose of the RPV is to provide information on the availability and functioning of primary healthcare services for statistics, development, and planning purposes (FIHW, 2025). The RPV was expanded in 2020 to include private and occupational healthcare visits (FIHW, 2025), which has resulted in an increase in the number of registered primary healthcare visits (Online Supplement 1). For this reason, the annual data in the RPV are not directly comparable across years. As data from private and occupational healthcare providers are missing before 2020, the number of primary healthcare visits shows an increase from 2020 onwards. The diagnosis data are automatically collected from patient records into the RPV. The accuracy of the register depends on the quality and correctness of the information provided by the healthcare service provider; thus, the data may include missing diagnoses and duplicates. However, the FIHW may contact the healthcare service provider if any quality issues are identified. As our aim was to study potential discrepancies in the registered need for services, the data were not further processed. More information on the data, their processing, and quality is available from the FIHW (https://thl.fi/en/statistics-and-data/information-on-statistics/description-of-statistics/primary-health-care).

We extracted diagnosis data from the RPV for mental disorders (F00-F99), circulatory system diseases (I00-I99), respiratory system diseases (J00-J99), and musculoskeletal system diseases (M00-M99) and formed diagnosis rate trajectories for the years 2018-2024 (FIHW, 2025). These diagnostic categories were selected because they represent the largest groups in Finnish primary healthcare (Lehto *et al.* 2024) and encompass both chronic and acute conditions, capturing a broad spectrum of diseases. Additionally, for each diagnostic group, the counts of a single diagnosis (F20 schizophrenia, I10 essential hypertension, J45 asthma, M17 knee arthrosis) were extracted for further analysis. The diagnosis rate trajectories were constructed by plotting the number of diagnoses as a function of time. We retrieved the diagnosis counts for the entire country (population: 5.58 million), and for two neighbouring, differently sized well-being regions: North Ostrobothnia (population: 416,000) and Central Ostrobothnia (population size: 68,000).

### Statistical analyses

The annual national- and regional-level diagnosis numbers for the four diagnostic groups were presented for the period 2018–2024. Joinpoint regression analysis was used to assess trends and potential discrepancies in diagnosis rate trajectories across diagnostic groups from 2018 to 2024 (Kim *et al*., 2000). Two models were analysed: Model 1, which assumed a single linear trend with zero joinpoints, and Model 2, which allowed for one joinpoint to identify potential changes in trend. The statistical significance of joinpoints and annual percentage changes (APCs) were evaluated using permutation tests, with 95% confidence intervals (CIs) and p-values. Descriptive statistics were used to examine the counts of specific diagnoses (F20, I10, J45, and M17) in relation to the rates of physician visits in primary healthcare. Analyses were conducted using the Joinpoint Regression Program, Version 5.3.0.0. P-values <0.05 were considered statistically significant.

## Results

### National trends

**Figure 1.**
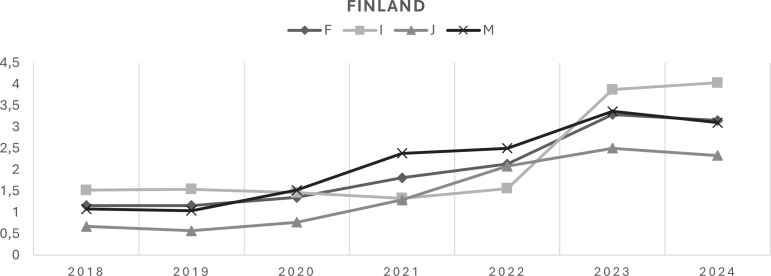
Diagnosis rate (in millions) trajectories (nationwide) for mental disorders (F00-F99), circulatory system diseases (I00-I99), respiratory system diseases (J00-J99), and musculoskeletal system diseases (M00-M99) from 2018 to 2024.

The overall trend (Model 1) showed a statistically significant increase in national-level diagnosis rates for all four diagnostic groups from 2018 to 2024 (Figure 1), with an APC of 21.9% (95% CI: 15.9–28.2) for mental disorders, 18.9% (95% CI: 1.1–39.9) for circulatory system diseases, 31.5% (95% CI: 8.8–59.8) for respiratory system diseases, and 24.0% (95% CI: 15.7–33.0) for musculoskeletal disorders. In Model 2, a statistically significant APC was observed only for circulatory system diseases in 2021–2024 (APC 50.8%, 95% CI: 29.7–92.8) (Table 1). Both linear and non-linear trends were observed in national-level diagnosis rate trajectories.

**Table 1.** The results of joinpoint regression analyses for national- and regional-level diagnosis rates across four diagnostic groups from 2018 to 2024

Variable	Model	Time period	APC	95% CI for APC	p-value
**Finland**					
F	Model 1	2018–2024	21.9	15.9 to 28.2	<0.001
	Model 2	2018–2020	10.9	-52.2 to 157.3	0.651
		2020–2024	26.6	-3.0 to 65.3	0.062
I	Model 1	2018–2024	18.9	1.1 to 39.9	0.041
	Model 2	2018-2021	-6.2	-25.8 to 9.0	0.436
		2021–2024	50.8	29.7 to 92.8	<0.001
J	Model 1	2018–2024	31.5	8.8 to 59.8	0.006
	Model 2	2018–2022	38.2	-12.2 to 117.6	0.092
		2022–2024	16.3	-72.3 to 388.6	0.696
M	Model 1	2018–2024	24.0	15.7 to 33.0	<0.001
	Model 2	2018–2022	30.1	-8.1 to 84.1	0.083
		2022–2024	9.9	-63.4 to 229.8	0.747
**North Ostrobothnia**					
F	Model 1	2018–2024	39.7	24.7 to 56.8	<0.001
	Model 2	2018–2022	49.4	-8.1 to 142.9	0.071
		2022–2024	18.1	-74.6 to 449.3	0.687
I	Model 1	2018–2024	28.5	17.5 to 40.6	<0.001
	Model 2	2018–2021	49.0	37.4 to 66.7	<0.001
		2021–2024	10.9	-0.7 to 20.0	0.057
J	Model 1	2018–2024	49.0	30.6 to 70.0	<0.001
	Model 2	2018–2022	71.6	63.6 to 83.3	<0.001
		2022–2024	4.6	-7.2 to 23.8	0.356
M	Model 1	2018–2024	45.0	21.7 to 73.0	0.003
	Model 2	2018–2021	92.5	64.2 to 148.0	<0.001
		2021–2024	9.2	-15.6 to 27.7	0.306
**Central Ostrobothnia**					
F	Model 1	2018–2024	37.9	24.3 to 53.1	<0.001
	Model 2	2018–2021	62.5	49.1 to 84.5	<0.001
		2021–2024	17.1	2.7 to 27.8	0.036
I	Model 1	2018–2024	16.5	10.6 to 22.9	<0.001
	Model 2	2018–2020	24.9	-5.0 to 64.3	0.073
		2020–2024	13.3	3.9 to 23.5	0.025
J	Model 1	2018–2024	20.8	9.1 to 33.8	0.005
	Model 2	2018–2020	-5.4	-26.2 to 29.5	0.695
		2020–2024	33.3	7.9 to 77.2	0.016
M	Model 1	2018–2024	21.1	16.1 to 26.3	<0.001
	Model 2	2018–2021	26.3	-4.3 to 66.6	0.069
		2021–2024	16.1	-12.0 to 53.2	0.146

*F* mental disorders, *I* circulatory system diseases, *J* respiratory system diseases, *M* musculoskeletal system diseases.

### Regional variations

**Figure 2.**
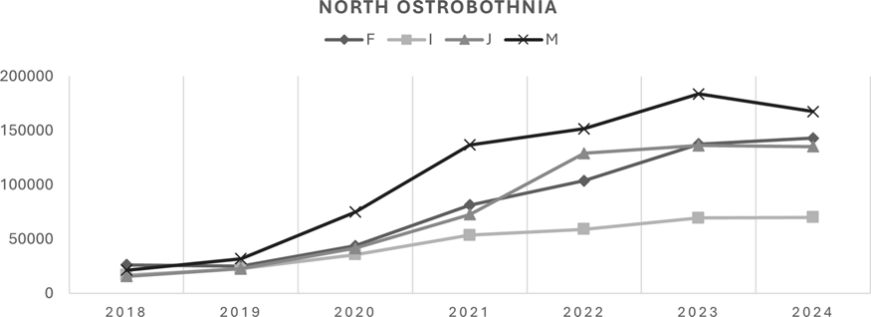
Diagnosis rate trajectories (North Ostrobothnia) for mental disorders (F00-F99), circulatory system diseases (I00-I99), respiratory system diseases (J00-J99), and musculoskeletal system diseases (M00-M99) from 2018 to 2024.

In North Ostrobothnia, a significant increase in diagnosis rates (Model 1) was observed across all diagnostic groups, with an APC of 49.0% (95% CI: 30.6–70.0) for respiratory system diseases, 45.0% (95% CI: 21.7–73.0) for musculoskeletal disorders, 39.7% (95% CI: 24.7–56.8) for mental disorders, and 28.5% (95% CI: 17.5–40.6) for circulatory system diseases (Figure 2). Model 2 showed significant increases in North Ostrobothnia for circulatory system diseases (APC 49.0%, 95% CI: 37.4–66.6) and musculoskeletal disorders (APC 92.5%, 95% CI: 64.2–148.0) in 2018–2021, and for respiratory system diseases (APC 71.6%, 95% CI: 63.6–83.3) in 2018–2022. Other APCs in Model 2 were not statistically significant.

**Figure 3.**
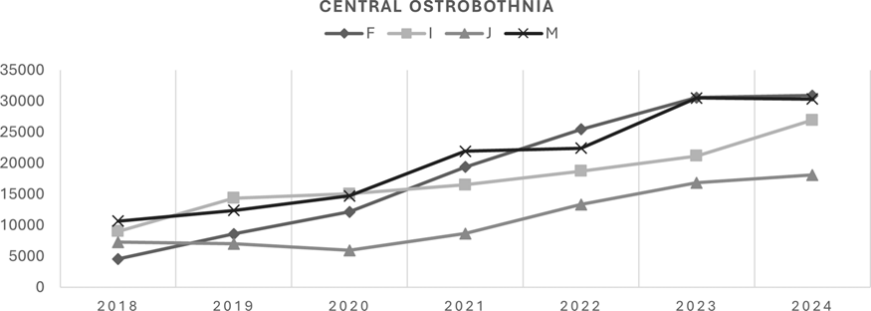
Diagnosis rate trajectories (Central Ostrobothnia) for mental disorders (F00-F99), circulatory system diseases (I00-I99), respiratory system diseases (J00-J99), and musculoskeletal system diseases (M00-M99) from 2018 to 2024.

In Central Ostrobothnia, diagnosis rates increased significantly across all diagnostic groups from 2018 to 2024 (Model 1), with APCs of 37.9% (95% CI: 24.3–53.1) for mental disorders, 16.5% (95% CI: 10.6–22.9) for circulatory system diseases, 20.8% (95% CI: 9.1–33.8) for respiratory system diseases, and 21.1% (95% CI: 16.1–26.3) for musculoskeletal disorders (Figure 3). In Model 2, statistically significant growth was observed for mental disorders in both 2018–2021 (APC 62.5%, 95% CI: 49.1–84.5) and 2021–2024 (APC 17.1%, 95% CI: 2.7–27.8), and for circulatory system diseases (APC 13.3%, 95% CI: 3.9–23.5) and respiratory system diseases (APC 33.3%, 95% CI: 7.9–77.2) in 2020–2024. Other APCs were not statistically significant.

Both linear and non-linear trends were found in regional-level diagnosis rate trajectories. The overall trend showed an increase in diagnosis rates at both national and regional levels for all specific diagnoses (F20, I10, J45, and M17) relative to the rates of physician visits (Online Supplement 1).

## Discussion

### Main findings

At both national and regional levels, the number of registered primary healthcare diagnoses has increased between 2018 and 2024, but the growth has not been uniform. We observed regional and diagnostic group-specific discrepancies in diagnosis rate trajectories.

### Trends in diagnosis rate trajectories

Model 1 indicated that primary healthcare diagnoses significantly increased between 2018 and 2024 at both the national and regional levels irrespective, of the diagnostic group. In addition, the numbers of individual diagnoses increased substantially; for example, the number of hypertension diagnoses more than doubled. The increase in the number of registered primary healthcare diagnoses suggests that the use of the RPV has increased in recent years. There are several possible explanations for this finding. First, the coverage of the RPV has naturally increased (García‑Velázquez *et al.,* 2022, Suokas *et al.,* 2024) due to the integration of visits to occupational health services and private healthcare into the register during the follow-up period (FIHW, 2025). Secondly, the social and healthcare reform may have accelerated the registration of diagnoses to maximize the funding of well-being regions through higher service need coefficients. Our analysis indicated increases in the rates of all specific diagnoses relative to physician visit rates. However, it should also be noted that the COVID-19 pandemic may have caused substantial variation in healthcare visits (Moynihan *et al.,* 2021 *)* and the registration of diagnoses during our study period.

There were differences in APCs, and the joinpoint regression variably suggested that either Model 1 or Model 2 with a joinpoint in 2021 or 2022 best fit the data, indicating that the number of diagnoses has not increased uniformly across all trajectories. The trends identified in Model 2 indicate national- and regional-level discrepancies in diagnosis rate trajectories. These discrepancies suggest differences in diagnosis registration practices across regions and healthcare sectors. Our results are consistent with a previous Finnish study reporting high discrepancies in registered physician visits among individuals with hypertension, asthma, diabetes, and mental disorders (García‑Velázquez *et al*., 2022). Our findings also support earlier criticisms regarding the inaccuracy of the RPV (Häkkinen *et al*., 2020; García‑Velázquez *et al*., 2022; Holster *et al*., 2022).

Compared to the national level, APCs were higher in the more populous Northern Ostrobothnia but mainly lower in the smaller Central Ostrobothnia in Model 1. In general, the more resources available, the better they can be allocated to the necessary activities, improving healthcare service quality (Mosadeghrad, 2014). It has been speculated that larger regions may have better capabilities than smaller well-being regions to register more diagnoses relative to the region’s morbidity, due to greater resources and more advanced technologies. However, since our data cover only two regions, no conclusions can be drawn, and broader data including more well-being regions will be needed in future studies to confirm these patterns. Regional differences in the enrolment of private and occupational healthcare providers into the register may also have contributed to variations observed in our study. Moreover, the use of different medical record systems between well-being regions may cause variation in the recording of the information across different areas (García‑Velázquez *et al*., 2022), leading to differences in the trajectories observed in our study.

### Strengths and limitations

The major strength of this study was the use of comprehensive register data, which enabled both national- and regional-level analyses over a six-year follow-up period. The application of joinpoint regression analysis allowed for the identification of changes in trends. Our study provides readily accessible results that can support healthcare decision-making.

Our dataset enabled us to examine diagnosis rate trajectories at a general level but did not allow for the analysis of potential causes or their magnitude. The RPV has been criticized for inaccuracies in the recording of diagnostic data (Häkkinen *et al*., 2020; García‑Velázquez *et al*., 2022; Holster *et al*., 2022), which may also lead to imprecision in our results. This is visible in our study, for example, in the sharp national-level increase in circulatory system diseases in 2022–2023, which is mainly explained by changes in the patient record system in large well-being regions in Southern Finland, leading to inconsistencies in those regions – particularly in the major diagnostic groups – since 2021 (FIHW, 2023). Nevertheless, as the same data is used as a tool for societal decision-making, our study allows for an evaluation at a similar level. As experiences from other countries show that developing an equitable funding system is a long-term process (Häkkinen *et al*., 2020), more studies with longer follow-ups are needed to confirm our findings. The relatively wide confidence intervals in our study primarily reflect variability in the trends over time and the flexibility of the joinpoint regression model. Allowing for a joinpoint to capture potential changes in trends introduces additional uncertainty in the annual percentage change estimates, which is reflected in wider confidence intervals.

### Clinical implications

For funding to be distributed equitably between and within well-being regions, the register-based need for services should evolve consistently, with changes driven by morbidity rather than practices (Smith, 2008). Poor timeliness and coverage of registers pose challenges to the validity of conclusions drawn (Ranstam *et al*., 2008). Variations in practices lead to unequal funding allocations between well-being regions and should be ignored when calculating need coefficients (Smith, 2008). Our findings suggest that standardizing diagnosis registration practices is needed to reduce discrepancies found in the current study. To address regional inequalities, stronger support for collecting diagnostic data should be provided to well-being regions to ensure comprehensive coverage and data accuracy.

## Conclusions

There are significant national and regional discrepancies in the registration of primary healthcare diagnoses in Finland. Since the same data is used at the national level to allocate healthcare funding, reducing these discrepancies by standardizing diagnosis registration practices is necessary to ensure equitable funding across well-being regions and diagnostic groups.

## Supporting information

Majuri and Pussinen supplementary material Majuri and Pussinen supplementary material

## Data Availability

The data is openly available from the FIHW (https://thl.fi/en/statistics-and-data/information-on-statistics/description-of-statistics/primary-health-care).

## Supplementary material

The supplementary material for this article can be found at https://doi.org/10.1017/S1463423625100625.
