# Supplementary material for: Discrepancies in the register of primary health care visits: a 6-year time series study from Finland
Source: Prim Health Care Res Dev. 2025 Nov 18;26:e95. doi: 10.1017/S1463423625100625 (PMC12646174; doi:10.1017/S1463423625100625)
Supplement: Majuri and Pussinen supplementary material [file S1463423625100625sup001.docx]

Online supplement 1.

Discrepancies in the Register of Primary Health Care Visits: A 6-year time series study from Finland

Online supplement table 1. National- and regional-level rates of physician visits in primary health care

|  | **2018** | **2019** | **2020** | **2021** | **2022** | **2023** | **2024** |
| --- | --- | --- | --- | --- | --- | --- | --- |
| Finland | 11 729 282 | 11 131 860 | 13 637 647 | 18 411 694 | 20 260 689 | 21 718 674 | 21 932 797 |
| North Ostrobothnia | 898 681 | 933 691 | 1 074 259 | 1 411 740 | 1 543 612 | 1 609 961 | 1 542 016 |
| Central Ostrobothnia | 137 414 | 132 364 | 146 726 | 183 324 | 189 186 | 204 671 | 197 047 |

Online supplement table 2. Rates of specific diagnoses per physician visit

|  | **2018** | **2019** | **2020** | **2021** | **2022** | **2023** | **2024** |
| --- | --- | --- | --- | --- | --- | --- | --- |
| Diagnosis rate per visit (% of visits) | % | % | % | % | % | % | % |
| **Finland** |  |  |  |  |  |  |  |
| F20 | 0.76 | 0.80 | 0.66 | 0.39 | 0.43 | 0.94 | 1.00 |
| I10 | 4.39 | 4.83 | 3.87 | 3.10 | 3.73 | 7.84 | 9.64 |
| J45 | 0.90 | 0.87 | 0.81 | 0.85 | 1.11 | 1.56 | 1.52 |
| M17 | 0.95 | 1.04 | 1.04 | 0.97 | 1.07 | 1.46 | 1.50 |
| **North Ostrobothnia** |  |  |  |  |  |  |  |
| F20 | 0.17 | 0.064 | 0.063 | 0.063 | 0.078 | 0.084 | 0.108 |
| I10 | 0.55 | 0.84 | 1.34 | 1.69 | 1.75 | 2.07 | 2.15 |
| J45 | 0.22 | 0.34 | 0.50 | 0.61 | 0.67 | 0.76 | 0.74 |
| M17 | 0.25 | 0.39 | 0.64 | 0.77 | 0.81 | 0.88 | 0.84 |
| **Central Ostrobothnia** |  |  |  |  |  |  |  |
| F20 | 0.064 | 0.100 | 0.068 | 0.070 | 0.067 | 0.114 | 0.275 |
| I10 | 2.78 | 4.39 | 3.73 | 3.51 | 3.52 | 3.85 | 4.75 |
| J45 | 0.85 | 1.48 | 1.13 | 1.32 | 1.37 | 1.56 | 1.67 |
| M17 | 0.89 | 1.10 | 1.08 | 1.03 | 1.14 | 1.61 | 1.71 |

*F20* schizophrenia, *I10* essential hypertension, *J45* asthma, *M17* knee arthrosis
